# Magnetically Separable MoS_2_/Fe_3_O_4_/nZVI Nanocomposites for the Treatment of Wastewater Containing Cr(VI) and 4-Chlorophenol

**DOI:** 10.3390/nano7100303

**Published:** 2017-09-30

**Authors:** Haijiao Lu, Jingkang Wang, Hongxun Hao, Ting Wang

**Affiliations:** 1 National Engineering Research Center of Industry Crystallization Technology, School of Chemical Engineering and Technology, Tianjin University, Tianjin 300072, China; luhaijiao@tju.edu.cn (H.L.); jkwang@tju.edu.cn (J.W.); wangting@tju.edu.cn (T.W.); 2 Collaborative Innovation Center of Chemical Science and Engineering, Tianjin 300072, China

**Keywords:** nZVI, modification, nanocomposites, Cr(VI), 4-CP, Fenton reaction, pollution treatment, recyclability, wastewater

## Abstract

With a large specific surface area, high reactivity, and excellent adsorption properties, nano zerovalent iron (nZVI) can degrade a wide variety of contaminants in wastewater. However, aggregation, oxidation, and separation issues greatly impede its wide application. In this study, MoS_2_/Fe_3_O_4_/nZVI nanocomposites were successfully synthesized by a facile step-by-step approach to overcome these problems. MoS_2_ nanosheets (MNs) acted as an efficient support for nZVI and enriched the organic pollutants nearby, leading to an enhanced removal efficiency. Fe_3_O_4_ nanoparticles (NPs) could not only suppress the agglomeration and restacking of MNs, but also facilitate easy separation and recovery of the nanocomposites. The synergistic effect between MNs and Fe_3_O_4_ NPs effectively enhanced the reactivity and efficiency of nZVI. In the system, Cr(VI) was reduced to Cr(III) by nZVI in the nanocomposites, and Fe^2+^ produced in the process was combined with H_2_O_2_ to further remove 4-Chlorophenol (4-CP) through a Fenton reaction. Furthermore, the nanocomposites could be easily separated from wastewater by a magnet and be reused for at least five consecutive runs, revealing good reusability. The results demonstrate that the novel nanocomposites are highly efficient and promising for the simultaneous removal of Cr(VI) and 4-CP in wastewater.

## 1. Introduction

As a typical contaminant in wastewater, Cr is one of the most toxic heavy metals to humans and the environment, which widely exists in leather tanning, metallurgy, paint pigments, etc. [1,2]. Cr mainly occurs in two common oxidation states (Cr(III) and Cr(VI)) in the environment. The toxicity of Cr(III) species is one hundred times lower than that of Cr(VI) [3]. Therefore, converting Cr(VI) to Cr(III) is frequently applied as an efficient approach for Cr(VI) removal. As another group of common water pollutants, chlorophenols (CPs) can be found in wastewater from various industrial processes, such as tanning, manufacturing preservatives, pesticides, and so on [4,5]. They are listed as “priority toxic pollutants” by the US Environmental Protection Agency (EPA) due to their strong toxicity and poor biodegradability [6,7]. Therefore, substantial studies have been conducted to remove CPs from wastewater. Among various methods reported, the dechlorination of CPs is considered to be the most effective method because the CP pollutants (phenol) can be subsequently oxidized to CO_2_ and H_2_O and thus be completely removed [8]. In fact, heavy metals and organic pollutants often co-exist in industrial wastewater, and wastewater containing both Cr(VI) and 4-CP is common [9]. Therefore, it is of great significance to remove Cr(VI) and 4-CP simultaneously from wastewater.

In the past decades, nano zerovalent iron (nZVI) has attracted great attention due to its excellent performance in the removal of a wide range of contaminants, including halogenated organic compounds [10], nitroaromatic compounds [11], organic dyes [12], phenols [13], heavy metals [14], metalloids [15], and so on. In particular, many studies have demonstrated that nZVI can effectively remove Cr(VI) and CPs in wastewater [3,16,17]. With a small particle size, large surface area, and superior reactivity, nZVI exhibits enhanced capability in the catalytic removal of contaminants compared with normal-sized iron particles [18]. However, due to a large specific surface area and intrinsic magnetic interaction, nZVI is easy to aggregate and can be oxidized by surrounding media, resulting in a loss of mobility and reactivity [19]. What is more, nZVI also has the trouble of separation from the degraded system, which makes it difficult to recover and reuse nZVI [20]. These problems limit the application of nZVI to a certain degree. To overcome these problems, great efforts have been made to modify nZVI [21,22].

As a frequently used strategy, nZVI can be immobilized on a support, such as clays [23], polysaccharides [24], resins [25], membranes [26], grapheme [27], and so on. With a layered structure similar to that of graphene, MoS_2_ has attracted tremendous research interest in many fields, such as catalysis, solar cells, electronic devices, and energy storage [28,29]. MoS_2_ nanosheets (MNs) are composed of single or several layers of S-Mo-S sandwich layers stacked together by van der Waals interactions [30]. MNs possess many superior properties, including a large surface area and excellent chemical and thermal stability, which make them favorable for various applications [31]. In recent years, a wide variety of methods have been proposed to prepare MNs, for example, mechanical exfoliation [32], liquid exfoliation [33], chemical vapor deposition [34], the hydrothermal method [35], and so on. It has been used as an alternative of graphene to act as a support for nZVI for aqueous pollutants removal [36]. Apart from avoiding the aggregation of nZVI, MNs also have the capability to enrich the organic pollutants nearby, leading to a greater removal efficiency [37]. In addition, MoS_2_ can be decorated with Fe_3_O_4_ nanoparticles (NPs), which are low-cost, easy to fabricate, and environmentally friendly [38]. Fe_3_O_4_ NPs can not only suppress the agglomeration and restacking of MNs, but also facilitate easy separation and recovery of the nanocomposites [37]. It helps to improve the sustainability of wastewater treatment process, which is a significant research trend in recent years [39,40].

Although many researchers have done substantial studies on the preparation and application of nZVI, MNs, and Fe_3_O_4_ nanoparticles [18,21,32,33,34,35], to the best of our knowledge, magnetically separable MoS_2_/Fe_3_O_4_/nZVI nanocomposites (MFNNs) have not been reported for the simultaneous removal of Cr(VI) and 4-CP in wastewater. In this study, to enhance the mobility and reactivity of nZVI and facilitate its separation and recovery from the degraded system, MoS_2_/Fe_3_O_4_/nZVI nanocomposites were successfully synthesized by a facile step-by-step method. They exhibited high activity, convenient separation, and good recyclability. These properties demonstrate that MoS_2_/Fe_3_O_4_/nZVI nanocomposites could be highly effective and efficient for pollution treatment in wastewater.

## 2. Materials and Methods 

### 2.1. Materials

MoO_3_ (AR, ≥99.5%) was purchased from Kermel Chemical Technology Co. Ltd. (Tianjin, China). Thiourea (NH_2_CSNH_2_) (AR, ≥99.0%), FeCl_3_·6H_2_O (ACS), FeSO_4_·7H_2_O (AR, ≥99.0%), NaBH_4_ (≥98.0%), and ammonia solution (AR, 25–28%) were purchased from Aladdin Industrial Corporation (Shanghai, China). 4-chlorophenol (≥99.0%) was purchased from J&K Chemical Ltd. (Beijing, China). K_2_Cr_2_O_7_ (≥99.95%) and H_2_O_2_ solution (≥30.00%) were purchased from Guangfu Fine Chemical Research Institute (Tianjin, China). Isopropyl alcohol (≥99.7%), ethanol absolute (≥99.8%), and anhydrous methanol (HPLC) were purchased from Real&Lead Chemical Technology Co. Ltd. (Tianjin, China). 1,5-diphenylcarbazide (DPC) (AR) was purchased from Chemart Chemical Technology Co. Ltd. (Tianjin, China). Phosphoric acid (≥85.0%) and sulfuric acid (95.0–98.0%) were purchased from Yuxiang Chemical Technology Co. Ltd. (Tianjin, China). All the compounds were used without further purification. Deionized water was provided by Barnstead Nanopure Water Purifier (Thermo Fisher Scientific, Waltham, MA, USA) throughout this work.

### 2.2. Synthesis of MoS_2_ Nanosheets (MNs)

In a typical procedure, 0.4 g MoO_3_ and 6.35 g thiourea were mixed together, thoroughly ground, and loaded into a quartz boat. The boat was quickly pushed into the hot zone of the tube furnace after the furnace temperature had been stabilized at 850 °C under the atmosphere of nitrogen. Before the reaction, the furnace was flushed with N_2_ for 30 min to remove any traces of oxygen. After calcination at 850 °C for 3 h, the tube furnace was cooled down to room temperature, and the resulting black powder was then collected.

### 2.3. Synthesis of MoS_2_/Fe_3_O_4_ Nanocomposite (MFN)

MFN was synthesized by a hydrothermal method. A total of 90 mg MNs was dispersed into 60 mL deionized water and isopropyl alcohol (1:1, volume ratio). Then, the mixture was treated by mechanical agitation and sonication at the same time for 3 h. After that, 0.0506 g FeCl_3_·6H_2_O and 0.0261 g FeSO_4_·7H_2_O were added to the suspension and the obtained mixture was then vigorously agitated at 80 °C for 30 min. After adding 0.75 mL ammonia solution quickly, the suspension was transferred into a 100 mL Teflon-lined stainless steel autoclave, sealed tightly, and heated at 120 °C for 2 h. After cooling naturally, the black product was collected by centrifugation, washed with deionized water and ethanol several times, and dried in a vacuum oven at 60 °C for 6 h. 

### 2.4. Synthesis of MoS_2_/Fe_3_O_4_/nZVI Nanocomposites (MFNNs)

**Scheme 1:**
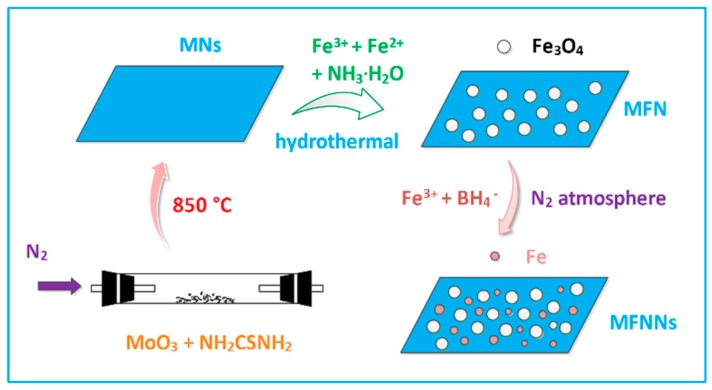
The synthesis schematic of MFNNs.

A total of 50 mg MFN was dispersed into 20 mL deionized water and isopropyl alcohol (1:1, volume ratio). After adding 0.0810 g FeCl_3_·6H_2_O, the obtained mixture was vigorously agitated under nitrogen atmosphere for 30 min. Then, 5 mL 0.18 M NaBH_4_ solution was added drop by drop at the speed of one to two drops per second into this mixture and vigorously stirred under nitrogen atmosphere. Subsequently, the suspension was agitated continuously for another 20 min. Finally, the black solid product was collected by centrifugation, washed with deionized water and ethanol several times, and dried in a vacuum oven at 60 °C for 6 h. The synthesis schematic of MFNNs is illustrated in Scheme 1.

### 2.5. Characterization

X-ray diffraction (XRD) patterns of the obtained solid products were obtained using a Rigaku D/max-2500 X-ray diffraction analyzer (Rigaku, Tokyo, Japan) with Cu Kα (λ = 0.154 nm) radiation at 40 kV/100 mA at a scanning speed of 6°/min. Nitrogen adsorption isotherms were measured with an ASAP 2020 adsorption analyzer (Micromeritics, Norcross, GA, USA) at 77 K. The Brunauer-Emmett-Teller (BET) method was utilized to calculate the specific surface areas. Scanning electron microscopy (SEM) images were taken by a Hitachi S4800 (Hitachi, Tokyo, Japan). High resolution transmission electron microscopy (HRTEM) and energy dispersive X-ray spectroscopy (EDS) measurements were performed on a Tecnai G2 F20 TEM (FEI, Eindhoven, The Netherlands) at an operating voltage of 200 kV. X-ray photoelectron spectroscopy (XPS) was performed on a Thermo ESCALAB 250XI (Thermo Fisher Scientific, Waltham, MA, USA) for compositional and chemical states analysis on the surface. The concentration of Cr(VI) was obtained by 1,5-diphenylcarbazide (DPC) method with a UV-Vis spectrophotometer (Hitachi, U-3010, Tokyo, Japan) at 540 nm (method detection limit (MDL) 0.31 mg/L, relative standard deviation (RSD) 3.9%). The concentration of 4-CP in samples was determined using high performance liquid chromatography (HPLC) (Agilent 1200LC, Santa Clara, CA, USA) equipped with a ZORBAX Eclipse Plus C18 column (Agilent, 4.6 × 250 mm, 5 μm, Santa Clara, CA, USA) and a UV-Vis detector at 280 nm (Agilent, MDL 0.83 mg/L, RSD 3.0%, Santa Clara, CA, USA). 

### 2.6. Simultaneous Removal of Cr(VI) and 4-CP

All experiments were carried out in 250 mL flasks containing 100 mL simulated wastewater (deionized water with 10–40 mg/L Cr(VI) and 20–120 mg/L 4-CP). The flasks were agitated at the speed of 400 rpm at 25 °C. A certain amount of MFNNs was added into the solution and the reaction was then timed immediately. After 15 min, H_2_O_2_ solution was added into the system. In the process, 1 mL of sample was withdrawn with a glass syringe at an interval of 5 min, and was then filtered through a 0.22 μm membrane filter. The concentrations of remaining Cr(VI) and 4-CP were detected by a UV-Vis spectrophotometer and HPLC, respectively. After the given reaction time of 60 min, the nanocomposites were separated by an external magnet and washed by the mixture of water and ethanol (1:1, volume ratio) three times. Furthermore, after drying up, the nanocomposites were reused in the next cycle of the removal experiment. Their reusability was evaluated by the removal efficiency of Cr(VI) and 4-CP over five cycles. All experiments were undertaken in triplicate. The average value of three experiments was used to evaluate the results. The relative standard deviation of the experimental results is about 5.1%.

## 3. Results and Discussion

### 3.1. Characterization

#### 3.1.1. XRD

**Figure 1:**
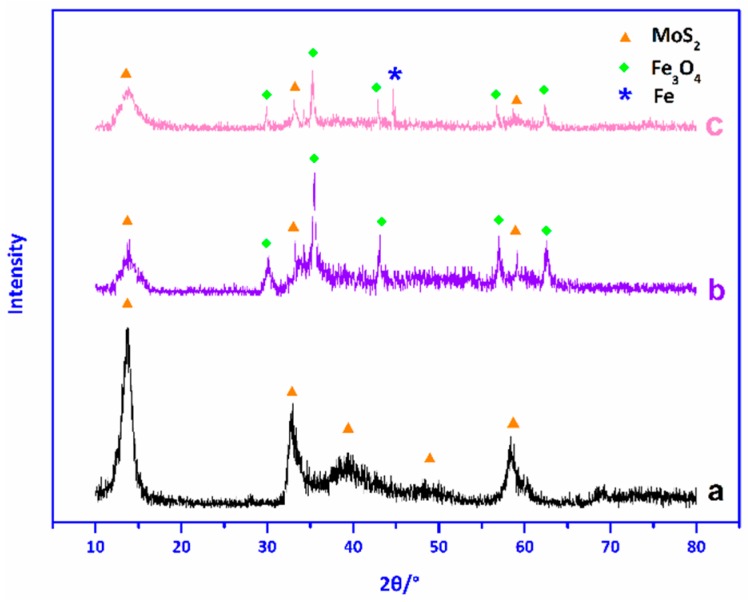
XRD patterns of (**a**) MoS_2_ nanosheets (MNs), (**b**) MoS_2_/Fe_3_O_4_ nanocomposite (MFN), (**c**) MoS_2_/Fe_3_O_4_/nZVI nanocomposites (MFNNs).

The XRD patterns of MNs, Fe_3_O_4_, MoS_2_/Fe_3_O_4_ nanocomposite (MFN), and the as-prepared MoS_2_/Fe_3_O_4_/nZVI nanocomposites (MFNNs) are shown in Figure 1. For the XRD pattern of MNs, the characteristic peaks of hexagonal MoS_2_ (JCPDS card No. 37-1492) at 2θ = 14.2°, 32.8°, 39.6°, 49.4°, and 58.5° could be found, which were assigned to the reflection of (001), (100), (103), (105), and (110) crystallographic planes, respectively [41]. In the XRD pattern of MFN, five diffraction peaks located at 2θ = 30.2°, 35.8°, 43.3°, 57.2°, and 62.6° were attributed to the planes of (220), (311), (400), (511), and (440) of Fe_3_O_4_ (JCPDS card No. 74-0748), respectively [42]. As shown in Figure 1, the relative intensity of diffraction peaks of MoS_2_ decreased to some extent due to the introduction of Fe_3_O_4_ NPs. Although the diffraction peaks at 2θ = 39.6° and 49.4° disappeared, the characteristic diffraction peaks of MoS_2_ at 2θ = 14.2°, 32.8° and 58.5° still remained or shifted slightly, indicating that the structure of MoS_2_ was maintained well in the preparation of the MFN. As depicted in the XRD pattern of MFNNs, the peak at 2θ = 44.7°demonstrated the (110) plane of Fe (JCPDS card No. 06-0696). Seen from the XRD patterns of the MFN nanocomposite and the as-prepared MFNNs, the synthesis of nZVI did not significantly affect the structure of MFN. Moreover, all the components (MoS_2_, Fe_3_O_4_, and nZVI) in the nanocomposites were well crystallized. Good crystallization of each component indicates that their respective uniformity is good. It can help to make the properties (such as activity and stability) of the whole nanocomposite more uniform, which will help to ensure the reproducibility and stability of the degradation process.

#### 3.1.2. BET

**Figure 2:**
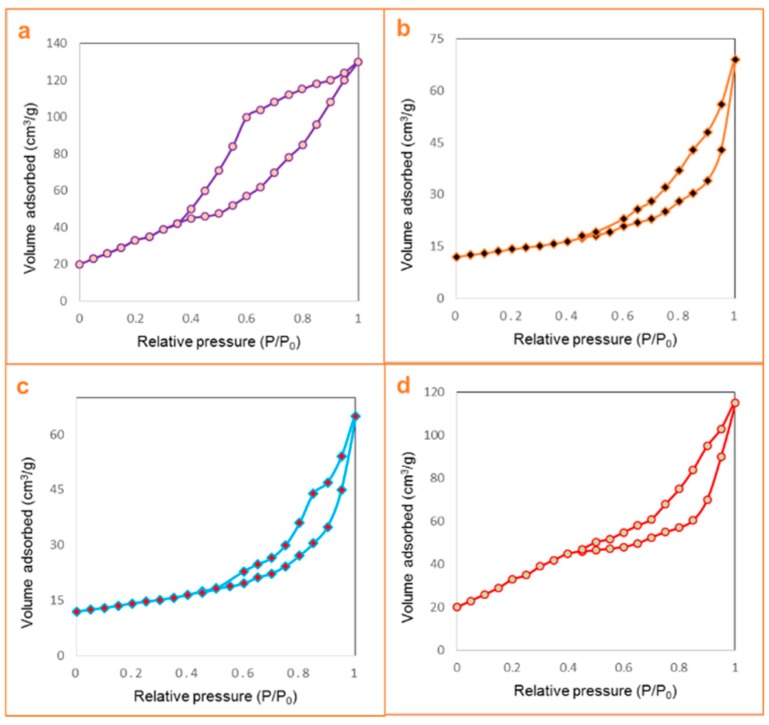
Nitrogen adsorption-desorption curves of (**a**) MNs, (**b**) Fe_3_O_4_ NPs, (**c**) bare nZVI, and (**d**) MFNNs.

To study the surface areas of MFNNs, nitrogen adsorption and desorption measurements were performed. The nitrogen adsorption-desorption curves of the MNs, Fe_3_O_4_ NPs, bare nZVI, and as-prepared MFNNs are shown in Figure 2, respectively. The BET surface areas of MNs, Fe_3_O_4_ NPs, bare nZVI, and as-prepared MFNNs were calculated to be 122.1, 42.2, 39.8, and 76.9 m^2^/g, respectively. It is evident that the specific surface area of MFNNs was much larger than that of bare nZVI. As expected, MNs significantly increased the specific surface area of nZVI. As Table 1 shows, the specific surface area of MFNNs was larger than most nZVI nanocomposites reported before. Moreover, homogeneous MoS_2_ nanosheets can be easily prepared in this study and the reproducibility of the process is good. Besides, the introduction of Fe_3_O_4_ as a spacer can isolate MoS_2_ from aggregation, thereby further increasing the specific surface area of the nanocomposites. This can facilitate the adsorption and enrichment of the contaminants nearby and thus greatly enhance the reaction activity to deal with contaminants [37].

#### 3.1.3. SEM

**Figure 3:**
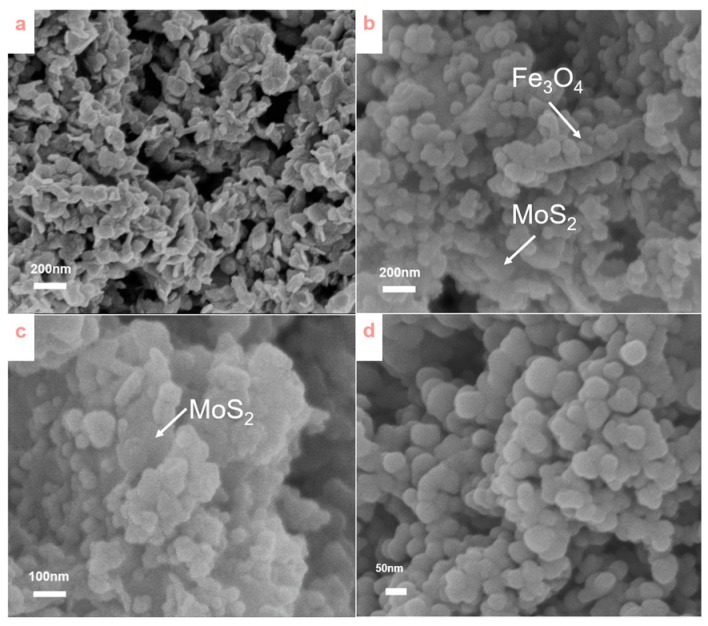
SEM images of (**a**) MNs, (**b**) MFN, (**c**) MFNNs, and (**d**) bare nZVI.

The morphologies of the products above were investigated by SEM. As can be seen from Figure 3a, the obtained MoS_2_ was mainly comprised of nanosheets with a uniform lateral size of about 100 nm, which was consistent with the study of Zhang et al. [48]. Figure 3b showed that magnetic Fe_3_O_4_ NPs were uniform in shape and size (about 60 nm) and well anchored on the surface of MNs. It is worth mentioning that by the synthesis method described in this study, MNs could not only provide a support for the growth of magnetic Fe_3_O_4_ NPs, but also inhibit their aggregation [49]. On the other hand, Fe_3_O_4_ NPs can also act as spacers between the MNs, preventing them from restacking in most solvents and thus improving their dispersity [38]. In addition, Fe_3_O_4_ NPs may also optimize the hydrophilicity and thus the dispersity of MNs. Contact angles of these materials have been tested to figure out their hydrophilicity. The results showed that the contact angles of Fe_3_O_4_ NPs, MNs, and MFNNs in our study were 75.5°, 94.0°, and 87.0°, respectively. A lower water contact angle means a higher surface energy, better wettability, and thus stronger hydrophilicity. Therefore, a contact angle measurement can provide evidence that Fe_3_O_4_ NPs could optimize the hydrophilicity and thus the dispersity of MNs. MFN obtained in this study could be well dispersed in water after sonication. Owing to this favorable property, MFNNs were then easily prepared in water by the borohydride reduction method. Seen from Figure 3c, NPs (about 50–60 nm) were well dispersed on the surface of MNs. MNs could protect nZVI NPs from surface oxidation and act as a support matrix for better dispersity. However, it is difficult to distinguish nZVI from Fe_3_O_4_ NPs due to their similar shape and size. According to Figure 3d, the size was 60–80 nm for bare nZVI NPs synthesized by the same method. The size of nZVI in MFNNs was smaller than that of bare nZVI, which might be attributed to the space restriction of the MFN. However, their coexistence can be illustrated by the XRD patterns mentioned above and further confirmed by TEM-EDS and XPS analysis in the following sections.

#### 3.1.4. TEM-EDS

**Figure 4:**
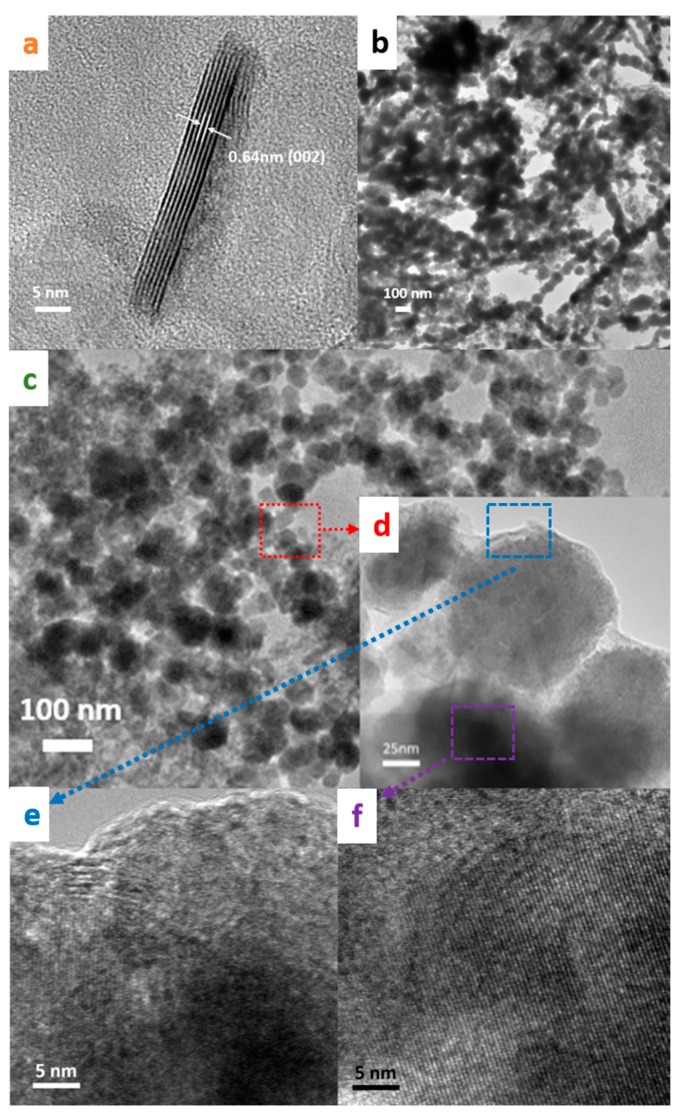
TEM images of (**a**) MNs, (**b**) bare nZVI, (**c**) MFNNs, (**d**) the red rectangular region in Figure 4c at a higher magnification, (**e**) the blue rectangular region in Figure 4d at a higher magnification, (**f**) the purple rectangular region in Figure 4d at a higher magnification.

To further examine the morphologies and crystalline structures of the products above, TEM characterization was also conducted. As shown in Figure 4a, MNs had a well-layered structure, with an interlayer spacing of 0.64 nm corresponding to the (002) plane of hexagonal MoS_2_. The TEM image of bare nZVI is shown in Figure 4b. It can be seen that bare nZVI NPs suffered from the problem of aggregation and formed chains which would result in the loss of dispersity, mobility, and specific surface area. Therefore, MFNNs were designed to inhibit the aggregation of NPs by acting as a support and spacer. As shown in Figure 4c,d, NPs were dispersed evenly onto the MNs. Figure 4e,f show Fe_3_O_4_ NPs and nZVI at a higher magnification, respectively. In fact, many studies have reported the preparation of Fe_3_O_4_/MoS_2_ nanocomposites in different routes and their results also showed that Fe_3_O_4_ NPs tend to disperse evenly onto the MNs [50,51,52]. However, the reason for the uniform distribution of Fe_3_O_4_ NPs on MoS_2_ is seldom discussed. Our hypothesis can be stated as follows. Iron ions are of an 18 electron configuration and easy to polarize. Thus, they have the tendency to form a covalent bond with S^2−^, which belongs to the soft base and is also easy to polarize. Since S^2−^ is evenly distributed in the MoS_2_ nanosheets, the interactions between S^2−^ and the bonding iron ions will facilitate to improve the uniformity of Fe_3_O_4_ NPs on MoS_2_ nanosheets. The study of Harris and Szilagyi [53] can support our explanation to a certain degree.

**Figure 5:**
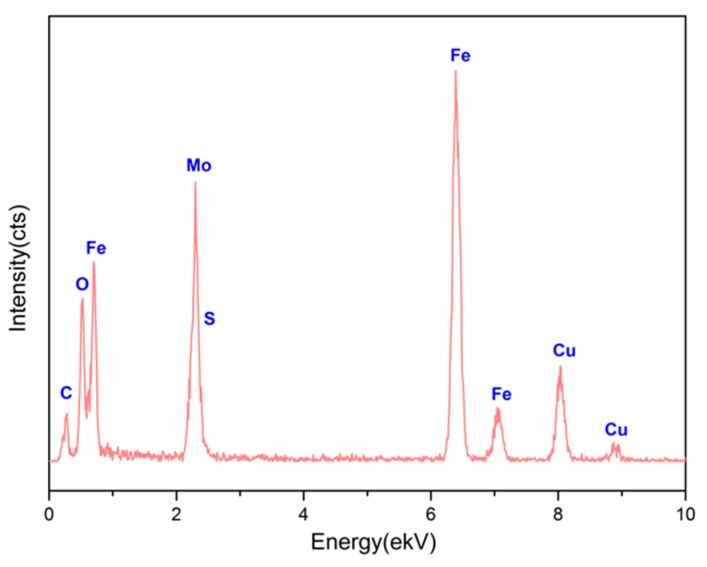
EDS of MFNNs in the region of Figure 4c.

Besides, elemental components analysis was also performed by an energy-dispersive X-ray spectrometer (EDS) equipped in TEM. As depicted in Figure 5, elemental peaks of Mo, S, Fe, and O were clear with C and Cu peaks coming from the grid. Furthermore, quantitative analysis revealed that the weight ratio of Mo, S, Fe, and O was about 8.81:7.79:5.53:1, which was close to the theoretical value of 8.97:7.90:5.41:1 (MoS_2_:Fe_3_O_4_:Fe = 5.36:1.30:1, weight ratio). Thus, the formation of both nZVI and Fe_3_O_4_ NPs could be verified. 

#### 3.1.5. XPS

**Figure 6:**
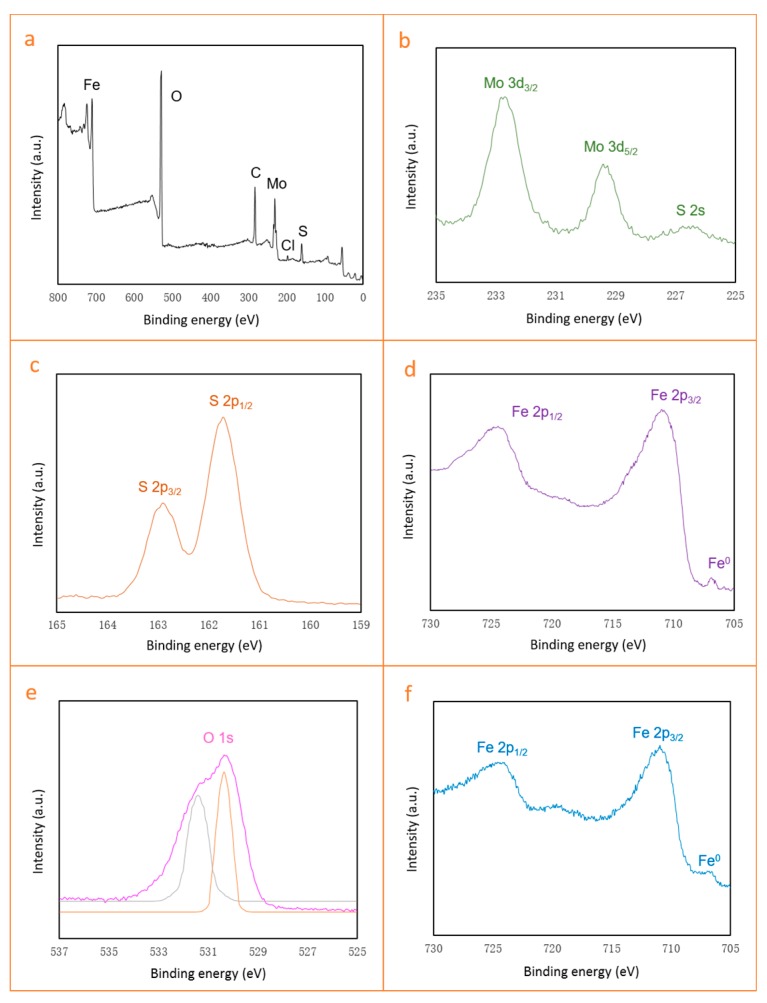
(**a**) The overall XPS spectrum of the MFNNs and individual (**b**) Mo 3d, (**c**) S 2p, (**d**) Fe 2p, and (**e**) O 1s XPS spectrums of the MFNNs, as well as (**f**) the Fe 2p XPS spectrum of bare nZVI.

As a versatile surface analysis technique, XPS is frequently used for compositional and chemical states analysis. Figure 6 showed the overall XPS spectrum of the MFNNs and individual Mo 3d, S 2p, Fe 2p, and O 1s XPS spectrums of the MFNNs, as well as the Fe 2p XPS spectrum of bare nZVI. As shown in Figure 6a, although the Cl element from the preparation processes also existed in MFNNs, its content was very low and was therefore negligible. The Mo 3d spectrum (Figure 6b) had two major peaks at 232.4 and 228.7 eV, corresponding to Mo 3d_3/2_ and 3d_5/2_ orbitals, respectively. The peaks for the S 2p_1/2_ and 2p_3/2_ orbitals of S^2−^ (Figure 6c) can be observed at 162.8 and 161.8 eV. The two distinct peaks at 724.6 and 710.9 eV corresponding to the Fe 2p_1/2_ and 2p_3/2_ orbitals (Figure 6d), as well as the peak at 530.1 eV corresponding to the O 1s orbital (Figure 6e), agreed well with the XPS of Fe_3_O_4_. The O1s spectrum of the nanocomposite could be decomposed into two peaks corresponding to Fe–O (530.0 eV) and hydroxyl groups (532.0 eV), respectively. Besides, no satellite peak at 719 eV could be seen, revealing that there was no Fe_2_O_3_, which could be formed by the surface oxidation of nZVI. Moreover, the peak at 706.9 eV of Fe^0^ (nZVI) was also very distinguished, demonstrating the high content of nZVI in MFNNs. By contrast, in the XPS of bare nZVI (Figure 6f), there was a satellite peak at 719 eV, implying that a layer of iron oxides (containing Fe_2_O_3_) definitely existed on the nZVI surface. As a consequence, the peak (at 706.3 eV) for Fe^0^ of bare nZVI was weak due to the limitation of XPS (less than 10 nm probing depth). In summary, the significant peaks corresponding to Fe_3_O_4_ and nZVI could verify the uniform loading of both of them on MNs. Furthermore, the comparison between nZVI on MNs and bare nZVI proved that MNs could effectively protect nZVI from surface oxidation and thus enhance its reactivity. 

### 3.2. The Composition of MFNNs

**Figure 7:**
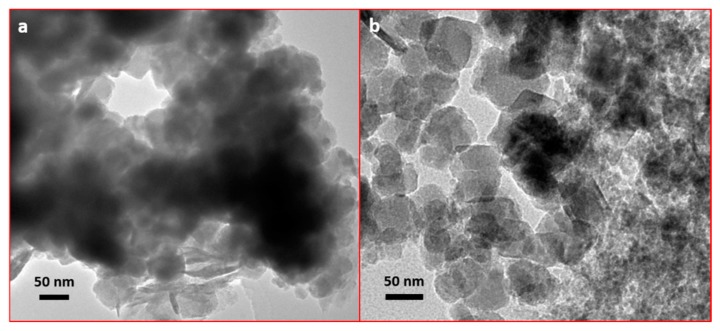
TEM images of (**a**) MFN loaded with excess Fe_3_O_4_ particles (MoS_2_:Fe_3_O_4_, weight ratio of 5:2), (**b**) MFNNs with excess free nZVI particles (MoS_2_:Fe_3_O_4_:nZVI, weight ratio of 4:1:1.15).

As the ratio of MoS_2_, Fe_3_O_4_, and nZVI will have a significant influence on the performance of MFNNs, it is of extreme importance to determine the optimum composition of MFNNs. The dosage of Fe_3_O_4_ introduced to MNs should be big enough to inhibit the aggregation of MNs and facilitate the separation of MFNNs. On the other hand, to ensure enough space for more nZVI particles to be loaded on MNs, the amount of immobilized Fe_3_O_4_ should be controlled. Figure 7a shows the TEM of MNs with overloaded Fe_3_O_4_ particles (MoS_2_:Fe_3_O_4_ weight ratio of 5:2), which was unfavorable for the following synthesis of MFNNs. After parallel experiments with different weight feed ratios of MoS_2_ and Fe_3_O_4_, the weight ratio of 4:1 was found to be the optimum value for this consideration. Therefore, the MoS_2_/Fe_3_O_4_ nanocomposite (weight ratio of 4:1) was chosen to further prepare MFNNs. Since nZVI is the reactive component in MFNNs for the treatment of contaminants, it is crucial to improve the ratio of nZVI to get a higher reactivity. However, the ratio should also be controlled below saturation. No free nZVI unbound to MFN should be observed. Otherwise, excess nZVI (relatively to MFN, see Figure 7b) will form aggregates. After trials, the weight ratio of 4:1:0.77 (MoS_2_:Fe_3_O_4_:nZVI) was eventually determined to be the optimum value for MFNNs. The typical synthesis process has been described in Section 2.2 and Section 2.3. 

### 3.3. Simultaneous Removal of Cr(VI) and 4-CP by Various Materials

**Figure 8:**
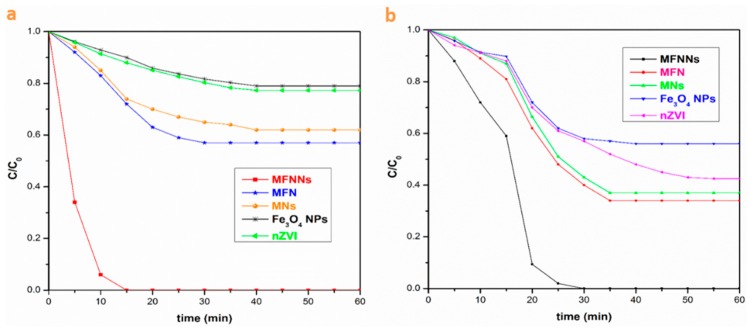
Effect of catalyst species on the simultaneous removal of (**a**) Cr(VI) and (**b**) 4-CP. (Experiment conditions: [Cr(VI)]_0_ = 10.0 mg/L, [4-CP]_0_= 100.0 mg/L,[catalyst]= 0.20 g/L, [H_2_O_2_]_0_ = 4.90 mM, pH = 3.0).

**Scheme 2:**
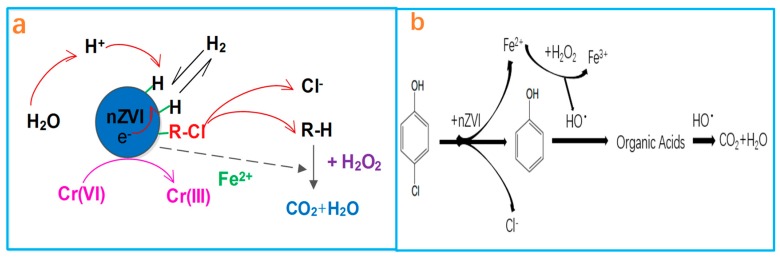
(**a**) Schematic of mechanisms for the simultaneous removal of Cr(VI) and 4-CP (R-Cl) by nZVI from aqueous solution. (**b**) Schematic of mechanisms for the degradation of 4-CP in this study.

In Figure 8, the performance of MNs, Fe_3_O_4_ NPs, bare nZVI, MFN, and MFNNs during the removal of Cr(VI) and 4-CP was demonstrated. The order of removal capacity for both Cr(VI) and 4-CP turned out to be MFNNs > MFN > MNs > nZVI > Fe_3_O_4_ NPs. It indicates that Cr(VI) could be completely removed by MFNNs in about 15 min. As mentioned above, H_2_O_2_ solution was added into the system at 15 min. Therefore, the added H_2_O_2_ was not consumed by Cr(VI) and could be used to oxidize 4-CP and its intermediates in the process. As Figure 8b shows, the addition of H_2_O_2_ led to a significant decrease of 4-CP by all the five materials. Particularly, since Fe^2+^ could be produced in the reduction of Cr(VI) by MFNNs and bare nZVI, the addition of H_2_O_2_ would form a Fenton system and thus greatly contribute to the further removal of 4-CP. The removal mechanism is illustrated in Scheme 2.

In addition, according to Figure 8b, 4-CP could be completely removed by MFNNs and H_2_O_2_ in about 30 min. In comparison, 4-CP could not be fully removed by other systems in 60 min, including bare nZVI. Without support, the removal efficiency of bare nZVI was very low due to the rapid formation of passive film by oxidation. MFN showed a higher efficiency than MNs, illustrating that the introduction of Fe_3_O_4_ NPs to MNs not only brought in magnetism, but also enhanced the adsorption capacity of MNs. This was mainly due to the above-mentioned synergistic effect between MNs and Fe_3_O_4_. More importantly, the removal efficiency of MFNNs was much higher than that of MNs, Fe_3_O_4_ NPs, and bare nZVI, proving the existence of a synergistic effect in this system. It has been previously reported that when coupling with Fe_3_O_4_ or support, nZVI can form numerous small batteries, accelerate the electron transform from Fe^0^ to Fe_3_O_4_, and then provide more reactive sites [54]. More active sites on the surface can promote the removal of Cr(VI) and 4-CP. The synergistic effects of MNs, Fe_3_O_4_, and nZVI all contributed to the high removal efficiency of Cr(VI) and 4-CP by MFNNs.

### 3.4. Effect of MFNNs Dosage

**Figure 9:**
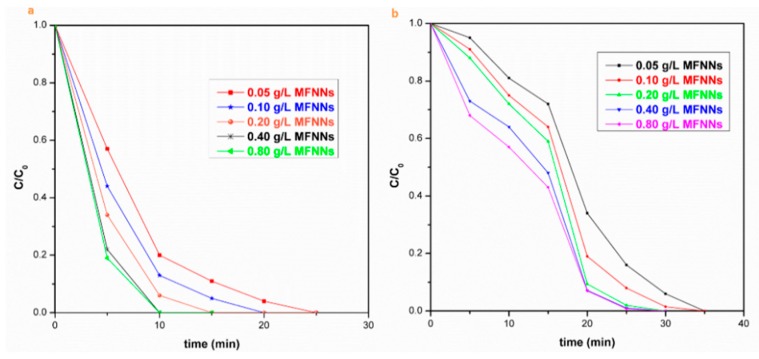
Effect of MFNN dosage on the simultaneous removal of (**a**) Cr(VI) and (**b**) 4-CP. (Experiment conditions: [Cr(VI)]_0_ = 10.0 mg/L, [4-CP]_0_ = 100.0 mg/L, [H_2_O_2_]_0_ = 4.90 mM, pH = 3.0).

The MFNN dosage was changed from 0.05 g/L to 0.80 g/L to study its effect on the removal of Cr(VI) and 4-CP. As shown in Figure 9a, the removal efficiency of Cr(VI) increased with the increment of MFNN dosage when it was below 0.40 g/L. No significant improvement could be seen when the dosage was further increased to 0.80 g/L. Meanwhile, according to Figure 9b, the removal of 4-CP before adding H_2_O_2_ was accelerated when the dosage of MFNN was increased from 0.05 g/L to 0.80 g/L. After the addition of H_2_O_2_ at 15 min, the removal rates of 4-CP first increased with the increase of MFNN dosage at the range of 0.05–0.20 g/L, and then showed no significant increment when the dosage was above 0.20 g/L. A higher MFNN dosage suggested more nZVI in the system, facilitating the removal of Cr(VI) and 4-CP by reduction and thus produced more Fe^2+^ to form a Fenton system with H_2_O_2_. However, as shown in Equation (1), too much Fe^2+^ can consume ·OH, impeding a further increase of 4-CP removal by a Fenton reaction [12]. Therefore, to achieve a high removal efficiency and reduce the cost, the MFNN dosage should be controlled.

(1) $$Fe^{2+}+\cdot OH\underset{}{\to }Fe^{3+}+OH^{-}$$

### 3.5. Effect of Contaminant Concentrations

**Figure 10:**
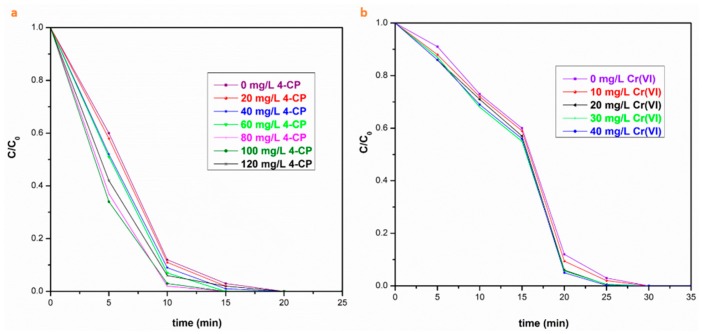
(**a**) Effect of initial 4-CP concentration on Cr(VI) removal by MFNNs. (Experiment conditions: [Cr(VI)]_0_ = 10.0 mg/L, [MFNNs] = 0.20 g/L, [H_2_O_2_]_0_ = 4.90 mM, pH = 3.0). (**b**) Effect of initial Cr(VI) concentration on 4-CP removal by MFNNs. (Experiment conditions: [4-CP]_0_ = 100.0 mg/L, [MFNNs] = 0.20 g/L, [H_2_O_2_]_0_ = 4.90 mM, pH = 3.0).

To evaluate the effect of the initial concentrations of Cr(VI) and 4-CP on the removal of each other, further studies were carried out by changing their initial concentrations. According to Figure 10a, when the initial concentration of 4-CP was increased from 0 mg/L to 40 mg/L, 100% removal of Cr(VI) took about 20 min. When it was further increased (60–100 mg/L), the time for 100% removal of Cr(VI) was shortened to 15 min. When it reached 120 mg/L, about 20 min was needed. It means that the reaction rates of Cr(VI) increased when the concentration of 4-CP was increased from 0 mg/L to 60 mg/L, and kept nearly constant when the concentration of 4-CP was increased from 80 mg/L to 100 mg/L, and decreased when the concentration of 4-CP was increased from 100 mg/L to 120 mg/L. The reason for this might be that Cl^−^ produced in the reduction of 4-CP could facilitate the conversion of Cr(VI) to Cr(III), but too much 4-CP would compete with Cr(VI) to react with nZVI and thus lower the reaction rate of Cr(VI) and nZVI. Figure 10b shows that when the concentration of Cr(VI) was increased from 0 mg/L to 20 mg/L, the time for 100% removal of 4-CP could be shortened from 30 min to 25 min. Further increasing the concentration of Cr(VI) to 30 mg/L or 40 mg/L had no influence on the removal rate of 4-CP. More Cr(VI) would react with more nZVI and thus produce more Fe^2+^ which could combine with H_2_O_2_ to form a Fenton system to degrade 4-CP and its intermediates in a shorter time [55]. However, due to the limited amount of H_2_O_2_, the removal rate of 4-CP nearly remained unchanged when the concentration of Cr(VI) was further increased to 30 mg/L or 40 mg/L. Therefore, Figure 10 demonstrates that when the initial concentrations of Cr(VI) and 4-CP were appropriate, their removal could be promoted mutually.

### 3.6. Effect of pH

**Figure 11:**
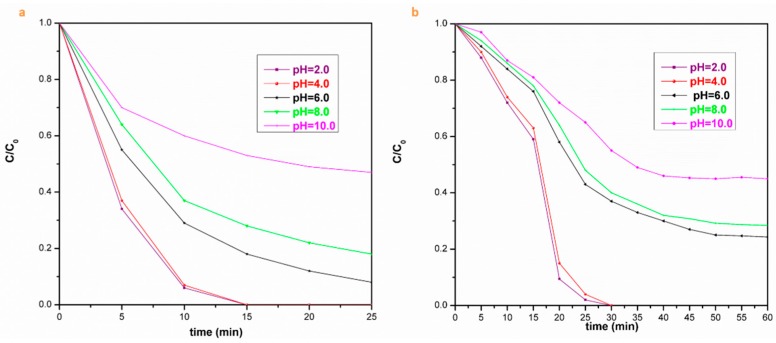
Effect of pH on the simultaneous removal of (**a**) Cr(VI) and (**b**) 4-CP by MFNNs. (Experiment conditions: [Cr(VI)]_0_ = 10.0 mg/L, [4-CP]_0_ = 100.0 mg/L, [MFNNs] = 0.20 g/L, [H_2_O_2_]_0_ = 4.90 mM).

The effect of pH was also experimentally investigated at pH = 2.0, 4.0, 6.0, 8.0, and 10.0. It can be seen from Figure 11 that pH had a significant effect on the removal of both Cr(VI) and 4-CP. With the increase of pH, the reaction rate decreased. The results are consistent with the mechanisms shown in Scheme 2. Since pH can affect the surface charge of nanocomposites, a lower pH will lead to a more positive surface charge, making it easier to attract the negative Cr(VI). When the pH increases, the enhanced electrostatic repulsion between the negative surface of MFNNs and Cr(VI) ions will result in a lower removal efficiency [2]. Moreover, at a higher pH, Cr(III) tends to form Cr(OH)_3_, which will adsorb on the surface of MFNNs, affecting the performance of nZVI [12]. On the other hand, the dechlorination reaction process in Scheme 2 can be briefly described by Equation (2). A higher pH suggested a lower concentration of H^+^, which was unfavorable for the reaction. Besides, the optimal pH of Fenton reaction is 2–4 [56] since the oxidation potential of ·OH produced by H_2_O_2_ decreases with the increment of pH [57]. What is more, it had been previously reported that the surface oxidation of bare nZVI would become more prominent at a higher pH, leading to the decrease of its reactivity and reaction rate [54,58]. Therefore, acidic conditions are preferable for the removal of Cr(VI) and 4-CP in wastewater. The conclusion agrees well with other studies on wastewater containing Cr(VI) and 4-CP [9,59,60].

(2) $$Fe+H^{+}+RCl\underset{}{\to }Fe^{2+}+RH+Cl^{-}$$

### 3.7. Adsorption Isotherm and Kinetics

As illustrated in our paper, the degradation process of 4-CP consisted of two steps: (1) add MFNNs to dechlorinate 4-CP; (2) add H_2_O_2_ to completely oxidize the intermedium after dichlorination. It is less meaningful to study the adsorption isotherms and kinetics of 4-CP since the process consists of two steps with different mechanisms. In comparison, the removal of Cr(VI) is achieved by MFNNs alone. Thus, the adsorption isotherm and kinetics of Cr(VI) is studied. The equilibrium adsorption capacity *q_e_* (mg/g) of Cr(VI) is calculated by Equation (3).

(3) $$q_{e}=\frac{(c_{0}-c_{e})V}{m}$$

where *c*_0_ and *c_e_* are the initial and equilibrium concentrations (mg/L) of Cr(VI), respectively. *V* is the volume of wastewater (L) and *m* is the mass of the adsorbent used (g).

To assess the adsorption capacity of adsorbents and facilitate the understanding of the adsorption mechanism, an adsorption isotherm was investigated with two frequently used models—the Langmuir model (Equation (4)) which assumes ideal monolayer adsorption onto a surface with identical adsorption sites and the Freundlich model (Equation (5)) which is an empirical model for a heterogeneous surface possessing different adsorption sites [61,62].

(4) $$\frac{c_{e}}{q_{e}}=\frac{1}{bq_{m}^{}}+\frac{c_{e}}{q_{m}}$$

(5) $$\ln q_{e}=\ln K+\frac{1}{n}\ln c_{e}$$

where *q_m_* (mg/g) is the maximum adsorption capacity for monolayer coverage and *b* (L/mg) is a constant. Their values can be calculated from the slopes and intercepts of the linear plots of *c_e_*/*q_e_* versus *c_e_* for Equation (4), respectively. *K* (mg^1−(1/*n*)^ L^1/*n*^/g) is a constant related to the sorption capacity, and *n* is an empirical parameter related to surface heterogeneity. Their values can be calculated from Equation (5).

The calculated results are listed in Table 2. As a higher value of *R*^2^ indicates a better-fit model, the adsorption of Cr(VI) onto MFNNs turns out to follow the Langmuir model, suggesting monolayer adsorption. 

Adsorption kinetics can provide information on the adsorption mechanism and develop appropriate mathematical models to describe the interactions. In this study, pseudo first-order (Equation (6)) and pseudo second-order (Equation (7)) were used to fit the experimental data [61,62].

(6) $$\ln(q_{e}-q_{t})=\ln q_{e}-k_{1}t$$

(7) $$\frac{t}{q_{t}}=\frac{1}{k_{2}q_{e}^{2}}+\frac{t}{q_{e}}$$

where *q_e_* is the concentration of Cr(VI) adsorbed after equilibrium, *q_t_* is the concentration of Cr(VI) adsorbed in time *t*, *k*_1_ (min^−1^) is the pseudo first-order rate constant. *q_e_* and *k*_1_ can be calculated from the intercepts and slopes of the straight lines obtained from plotting ln(*q_e_* − *q_t_*) vs. *t*, respectively. *k*_2_ (g/(mg·min)) is the pseudo second-order rate constant. *q_e_* and *k*_2_ can be calculated from the intercepts and slopes of the straight lines obtained from plotting *t*/*q_t_* vs. *t*, respectively. 

The results are shown in Table 3. According to the values of *R*^2^, the adsorption of Cr(VI) by MFNNs can be considered to obey the pseudo first-order model. 

### 3.8. Effect of H_2_O_2_ Concentration 

**Figure 12:**
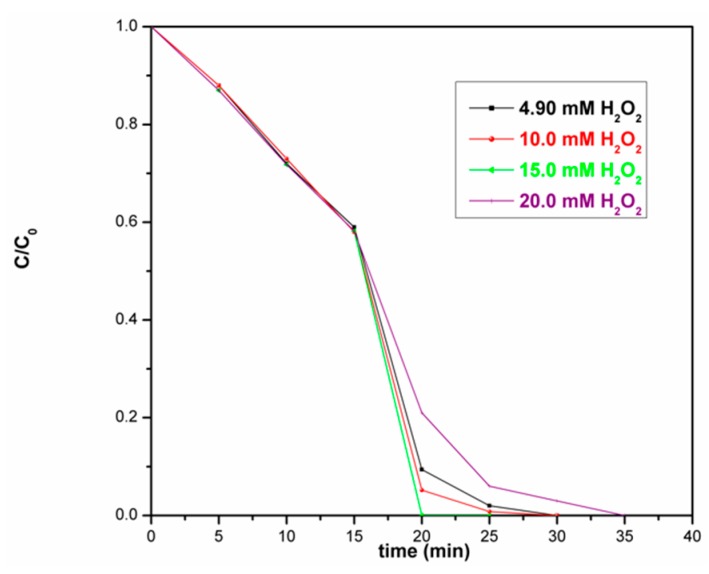
Effect of H_2_O_2_ concentration on 4-CP removal by MFNNs. (Experiment conditions: [Cr(VI)]_0_ = 10.0 mg/L, [4-CP]_0_ = 100.0 mg/L, [MFNNs] = 0.20 g/L, pH = 3.0).

Since H_2_O_2_ was added into the system at 15 min when Cr(VI) had been completely removed under the given experiment conditions, there was no need to study the effect of H_2_O_2_ concentration on Cr(VI) removal. As shown in Figure 12, the effect of H_2_O_2_ concentration on the removal of 4-CP was studied. With the increasing of H_2_O_2_ concentration, the removal efficiency of 4-CP increased first and then decreased. More H_2_O_2_ could produce more ·OH, promoting the Fenton reaction in the system. However, as shown in Equation (8), ·OH could also be consumed by excess H_2_O_2_, leading to the loss of removal efficiency [12]. Besides, MoS_2_ may be oxidized to MoO_3_ by excess H_2_O_2_, and thus the synergistic effect between MNs and Fe_3_O_4_ NPs would be hindered and the specific surface area of MFNNs would be reduced, leading to the decrease of MFNNs performance. Therefore, the concentration of H_2_O_2_ should be controlled at an appropriate range.

(8) $$H_{2}O_{2}+\cdot OH\underset{}{\to }H_{2}O+HO_{2}\cdot$$

### 3.9. Recyclability

**Figure 13:**
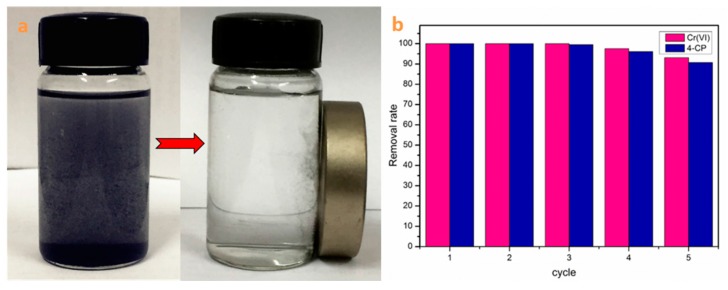
(**a**) Photographs of the nanocomposites in aqueous solution before (**left**) and after (**right**) the placement of a magnet. (**b**) The reusability of MFNNs for the removal of Cr(VI) in 15 min and 4-CP in 60 min. (Experiment conditions: [Cr(VI)]_0_ = 10.0 mg/L, [4-CP]_0_ = 100.0 mg/L, [MFNNs] = 0.20 g/L, [H_2_O_2_]_0_ = 4.90 mM, pH = 3.0).

Since MFNNs in this study were designed for convenient recovery by magnetic separation, their recovery and reusability were also investigated. In successive experiments, MFNNs were separated from wastewater by a magnet, washed with water and ethanol, and dried under vacuum. The results in Figure 13 show that MFNNs could be successfully recycled and reused for at least five consecutive runs with a removal rate of no less than 93% for Cr(VI) within 15 min and no less than 90% for 4-CP within 60 min, revealing excellent recycling and structural stability. Although nanomaterials based on nZVI have been put forward for the simultaneous removal of Cr(VI) and phenols, their separation and reuse have seldom been reported before [12,63,64].

## 4. Conclusions

In this study, novel MoS_2_/Fe_3_O_4_/nZVI nanocomposites were successfully synthesized by a facile step-by-step method and overcame the drawbacks of nZVI-oxidation, aggregation, and separation. Due to the synergistic effect between MoS_2_ nanosheets and Fe_3_O_4_ nanoparticles, the efficiency of MFNNs increased more than fivefold for the simultaneous removal of Cr(VI) and 4-CP in wastewater. Moreover, MFNNs could be successfully recycled and reused for at least five consecutive runs with a removal rate of no less than 93% for Cr(VI) within 15 min and no less than 90% for 4-CP within 60 min. Therefore, the novel MoS_2_/Fe_3_O_4_/nZVI nanocomposites hold great potential for the simultaneous removal of heavy metals and organic pollutants in wastewater treatment. 

## Figures and Tables

**Table 1:** Specific surface areas of different nZVI nanocomposites.

nZVI Nanocomposites	Specific Surface Area (m^2^/g)	Reference
MoS_2_/Fe_3_O_4_/nZVI	76.9	-
g-C_3_N_4_/MoS_2_/nZVI	37.5	[43]
nZVI/C yolk–shell particles	65.0	[44]
Fe_3_C/C/nZVI	42.3	[45]
activated carbon/nZVI	69.4	[46]
Mg(OH)_2_/nZVI	40.2	[47]

**Table 2:** Parameters of the Langmuir and Freundlich models for the adsorption of Cr(VI) on MFNNs.

Langmuir		Freundlich	
*q_m_* (mg/g)	346.34	*K* (mg^1−(1/*n*)^ L^1/*n*^/g)	0.103
*b* (L/mg)	0.013	*n*	1.64
*R*^2^	0.986	*R*^2^	0.919

**Table 3:** Parameters of the pseudo first and second-order kinetic models for the adsorption of Cr(VI) on MFNNs.

Pseudo First-Order		Pseudo Second-Order	
*q_e, cal_* (mg/g)	327.17	*q_e, cal_* (mg/g)	319.88
*k*_1_ (min^−1^)	0.123	*k*_2_ ((g/(mg·min))	0.0131
*R*^2^	0.990	*R*^2^	0.902
